# Supplementary material for: Patterns of Intron Gain and Loss in Fungi
Source: PLoS Biol. 2004 Nov 30;2(12):e422. doi: 10.1371/journal.pbio.0020422 (PMC532390; doi:10.1371/journal.pbio.0020422)
Supplement: Table S1 — Also available at http://genes.mit.edu/NielsenEtAl/. (4.3 MB ZIP). [file pbio.0020422.st001.zip › NielsenEtAl/html/321.html]

AN5152.1.NCU01814.1.MG06689.1.FG06003.1


```
 CLUSTAL W (1.82) Multiple Sequence Alignments - Introns Inserted


Sequence 1: MG06689.1	294 aa
Sequence 2: FG06003.1	234 aa
Sequence 3: NCU01814.1	278 aa
Sequence 4: AN5152.1	218 aa
Alignment Length: 301 aa
Number Identitical Residues: 83 aa
Alignment Score (without introns) 5343


MG06689.1 	--MSGFGNFG0PFCEGSTLPVCN0VLNKDNTGQRGGWGGCNLKGILLPNNQYLGNLG1VI
NCU01814.1	MGSTQFGNFH~VSSQ--------~--------RRRRWA---QKAPGSPYRNADSDSW~RL
FG06003.1 	MGSTEFGNFH0DFCRDSTLPVCN0LLSPKND-QNGDWGGCELKGISLSGGRHLGNLG~SI
AN5152.1  	----------~------------~---------------------------------~--
          	                                                            

MG06689.1 	MVCVVAIVVALYLLLR~SERKKAAVGRR2EMQLFLLGYIVIQICEIFTVGEISPLSETVR
NCU01814.1	HIYRTSVEIRLFLSAM2SERKRAAVGRR2EMQVFLLGYIVISICEIFTVGDF-PLNSTVR
FG06003.1 	LLAGIAIATAGFLLLR~SEKKRAAVGRR~EMQLFLLGYIIISICEIFSVGEF-PLNSTVR
AN5152.1  	----------------~--------MDR~EMQLFLLGYIIIEICEIFSVGGF-PLRNDVR
          	                           * ***:******:*.*****:** : ** . **

MG06689.1 	V0AFTGIHLGFIIATTWVLMLNAV~VGYQIIDDGTPLSLGLLVLSGLVLLIGTGYITLDT
NCU01814.1	I0AFTAIHIGFIIATTWILMLNAI~VGYQLLDDGTLLSLALIVGSAAILLVGTGYIALDT
FG06003.1 	V0AFSAIHIGMIIATCWILMLNAV0L----------------------------------
AN5152.1  	K0GFSAAHVAAITMTCWILLLNAL~VGFQFLDDGTPASIGLFLFSGLIFFIGTGYIALDT
          	  .*:. *:. *  * *:*:***: :. .  ...:  : .    :.     .:.  : .:

MG06689.1 	GFGWTGYWNESKED--YHHIALYVLYQLAPLIFIVAFFVLEAILVLRVLQETKPMI~YLV
NCU01814.1	GLHWTGYWDSSYELPKNRHIALYVLYQLAPLVFLVAYFVLEAILVIRILGETRPML~YLA
FG06003.1 	----------------------------VPLIFLVAYFVLEAILVVRILGETRPMI~YLA
AN5152.1  	AFDWTGEFATNEGN-GYRNIALYVLYQLFPLICLVAFFFLETFLVVRILGELRPML1YLS
          	.   :.    .         :     .  **: :**:*.**::**:*:* * :**: ** 

MG06689.1 	GAGLLFALGQIFNYVVSRYICNGTNGNIDGSLFQTIFTLASVVMVWIFWSSITEDDWPMP
NCU01814.1	SAAILFAAGQVFNYEVSKYICDGTSGKIDGALFETLFTLLAVVMNWVFWSSITEDDWPTV
FG06003.1 	AAGLLFAIGQVFNYAVSKYICDGTSGKIDGALFQTLFTLLSVIMVWVFWSSITEDDWPMP
AN5152.1  	AAGLLFAIGQIFNYVISTHLCQATDGKINGALFETLFTLVSVVMVWIFWSSITEDDWPTP
          	.*.:*** **:*** :* ::*:.*.*:*:*:**:*:*** :*:* *:***********  

MG06689.1 	VGSTYP---
NCU01814.1	PPNQFP---
FG06003.1 	VTNTYP---
AN5152.1  	MSQPMPGYN
          	  .  *. .
```
